# Surveillance Epidemiology and End Results Analysis Demonstrates Improvement in Overall Survival for Cervical Cancer Patients Treated in the Era of Concurrent Chemoradiotherapy

**DOI:** 10.3389/fonc.2015.00081

**Published:** 2015-04-13

**Authors:** Howard C. Hsu, Xiaochun Li, John P. Curtin, Judith D. Goldberg, Peter B. Schiff

**Affiliations:** ^1^Department of Radiation Oncology, New York University School of Medicine, New York, NY, USA; ^2^Department of Population Health, Division of Biostatistics, New York University School of Medicine, New York, NY, USA; ^3^Department of Obstetrics and Gynecology, Division of Gynecologic Oncology, New York University School of Medicine, New York, NY, USA

**Keywords:** cervical cancer, radiotherapy, chemotherapy, SEER, survival

## Abstract

**Background:**

In February 1999, the National Cancer Institute (NCI) issued a clinical alert based on five randomized trials that reported better overall survival (OS) with concurrent chemoradiotherapy (CCRT) than with surgery or radiation alone for locoregional cervical cancer. This study analyzes data from the surveillance epidemiology and end results (SEER) program to evaluate the improvement in survival in the era of CCRT.

**Methods:**

The SEER database was queried for FIGO stages IB2–IVA cervical cancer patients treated with radiotherapy between 1995 and 2002. Patients diagnosed between 1999 and 2002 (CCRT era) were assumed to have received CCRT more frequently than patients diagnosed between 1995 and 1998 (RT era). Cases were stratified by period of diagnosis, age, and SEER region. OS and cause specific survival (CSS) were compared between the two time periods with chi-square log-rank tests. Multivariable Cox models were also used to compare OS and CSS between the two time periods, with adjustment for stratification variables and other covariates.

**Results:**

The study included 3517 patients. Unadjusted OS and CSS were significantly improved in 1999–2002 compared with 1995–1998 (OS: *p* < 0.001, hazard ratio (HR): 0.81; CSS: *p* < 0.001, HR: 0.79). Significant improvements in OS and CSS were retained after adjustment for multiple variables (multivariable OS HR 0.78; CSS HR 0.76).

**Conclusion:**

Cervical cancer patients treated with radiotherapy after 1999 had improved OS and CSS compared with patients treated before 1999, likely reflecting increased usage of CCRT. This study adds to the population-level evidence supporting the adoption of CCRT as the standard of care for locoregional cervical cancer.

## Introduction

Prior to 1999, patients with cervical cancer were treated primarily with surgery or definitive radiation therapy. In February 1999, the National Cancer Institute (NCI) issued a clinical alert (1) based on five randomized clinical trials in locoregional cervical cancer that reported improved overall survival (OS) with concurrent chemoradiotherapy (CCRT) when compared with surgery or radiation alone (2–6). This resulted in the rapid adoption of CCRT for definitive treatment or selective adjuvant treatment for localized cervical cancer. In contrast, a clinical trial conducted by the NCI of Canada did not show a survival advantage with CCRT (7). However, three meta-analyses, including one from Canadian investigators, demonstrated improved OS and progression-free survival with CCRT (8–10).

This study seeks to confirm whether OS improved significantly in the United States after the NCI alert was issued (CCRT era). Our hypothesis is that the adoption of CCRT for locoregional cervical cancer in the United States was rapid after the NCI alert and resulted in an observable increase in **OS**. While chemotherapy usage was not recorded in the SEER*Stat analytical interface for the surveillance epidemiology and end results (SEER) database, we considered any rapid and significant change in survival rates around the time of the NCI alert to be indirect evidence of changes in chemotherapy usage because no other known changes occurred for the cervical cancer patient population during the study time period.

## Materials and Methods

### Data source

The SEER database is a NCI program that currently includes 18 regions. Since the data for Greater California (excluding San Francisco, Los Angeles, and San Jose), Kentucky, Louisiana, New Jersey, and Greater Georgia (excluding Atlanta and Rural Georgia) were not available for years prior to 2000, we excluded data from these four regions in our analyses. Therefore, 13 regions were included in our analyses. Compared to the general US population, the SEER population is similar with respect to poverty and education, but is somewhat more urban and has a higher proportion of foreign-born persons (11). SEER data (2011 submission) included cancer cases diagnosed up to and including year 2009, with a follow-up cutoff date of December 31, 2009. Chemotherapy usage was not recorded in the SEER*Stat analytical interface for the SEER database; so, it could not be incorporated explicitly into our analysis.

### Patient selection

SEER data were queried using SEER*Stat version 7.1.0 software (12). Patients were selected from the 13 regions that were included in both time periods using the following inclusion criteria: malignancy of the cervix uteri, FIGO stages IB2–IVA, diagnosis years 1995–2002, radiotherapy given, and no other malignancy. FIGO stages IB2–IVA were chosen to correspond with the patient populations allowed onto the randomized clinical trials. Stages II–IVA were recoded from the SEER Extent of Disease variable “EOD 10 – extent (1988–2003).” Stage IB2 included the subset of stage I patients with tumor size >4 cm. Only patients with cervical cancer as their sole malignancy were included to allow analysis of both **OS** and cause specific survival (CSS) (using the “SEER cause-specific death classification” variable) in the same cohort.

### Selection of time periods

Since the CCRT recommendation was made in 1999, we selected patients diagnosed in the four prior years (1995–1998) and the four subsequent years (1999–2002) for comparison. Patients diagnosed between 1995 and 1998 were presumed to have received radiotherapy and less frequently chemotherapy, while those diagnosed between 1999 and 2002 were presumed to have received CCRT more frequently.

### Statistical methods

Cases were stratified by period of diagnosis (1995–1998 and 1999–2002), age group, and SEER regions. Log-rank tests were used to test the heterogeneity within each of the two time periods. Chi-square tests were used to compare the distributions of the diagnostic characteristics between the two time periods. Log-rank tests were used to compare OS and CSS in 1999–2002 and 1995–1998 without adjustment for any other variables. Competing risk methods (“cmprsk” package, R software, Version 2.14) were used to compare CSS for 1995–1998 and 1999–2002, with other causes of death considered as competing risks to cervical cancer death. In addition, OS and CSS by the time periods were compared with log-rank tests stratified by age group and by SEER region. Multivariable Cox regression models were used to obtain hazard ratios (HRs) for OS and CSS for the time period comparison, with adjustment for age, region, and other covariates individually and jointly. Stepwise variable selection procedures were used to obtain the final models. SAS version 9.3 was used for all analyses. R version 2.13.1 was used to plot Kaplan–Meier survival curves.

## Results

The study included a total of 3517 patients from the 13 regions in the SEER database that had data spanning the entire study period. There were 1758 patients (50%) in 1995–1998 and 1759 patients (50%) in 1999–2002. Patients diagnosed in 1995–1998 had longer survival follow up (approximately 0–170 months) than those diagnosed in 1999–2002 (approximately 0–120 months) at the follow-up cutoff date of December 31, 2009.

**Figure 1:**
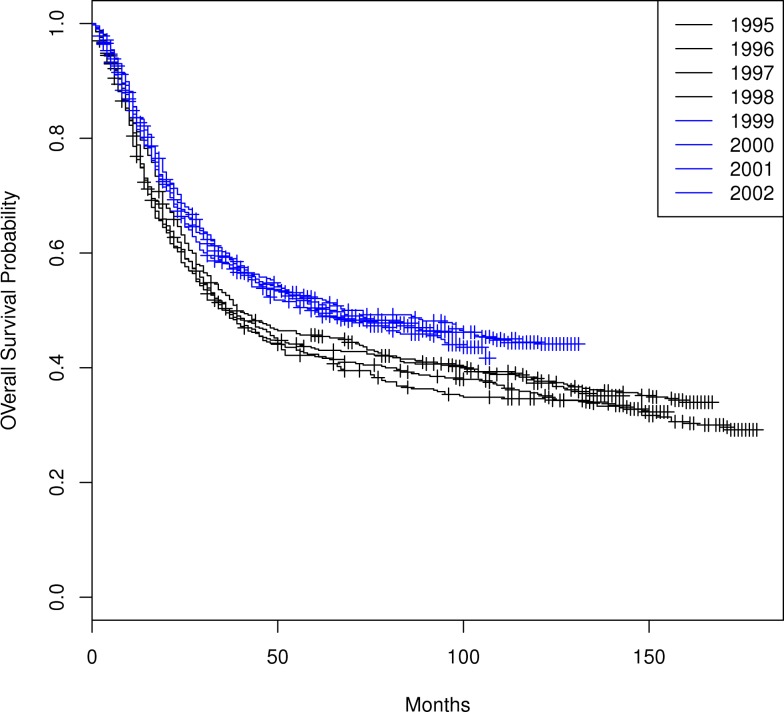
**Kaplan-Meier overall survival curves for individual years 1995–2002**.

The Kaplan–Meier OS curves (Figure 1) for each of the 8 years comprising the two time periods demonstrate a difference in OS between the two time periods (1995–1998 versus 1999–2002) but similarity in OS among the years within each time period. There is no significant heterogeneity within each of the two time periods (OS: 1995–1998 *p* = 0.69, 1999–2002 *p* = 0.88; CSS: 1995–1998 *p* = 0.69, 1999–2002 *p* = 0.68).

**Figure 2:**
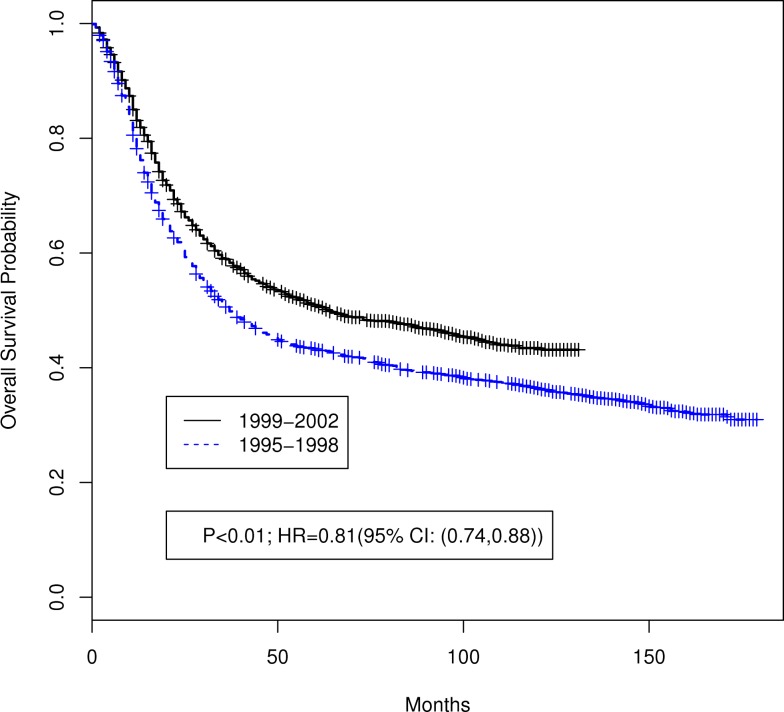
**Unadjusted Kaplan–Meier overall survival curves by 4-year time period (1999–2002 versus 1995–1998)**. Log-rank test *p* value and hazard ratio (HR) for death are shown.

**Figure 3:**
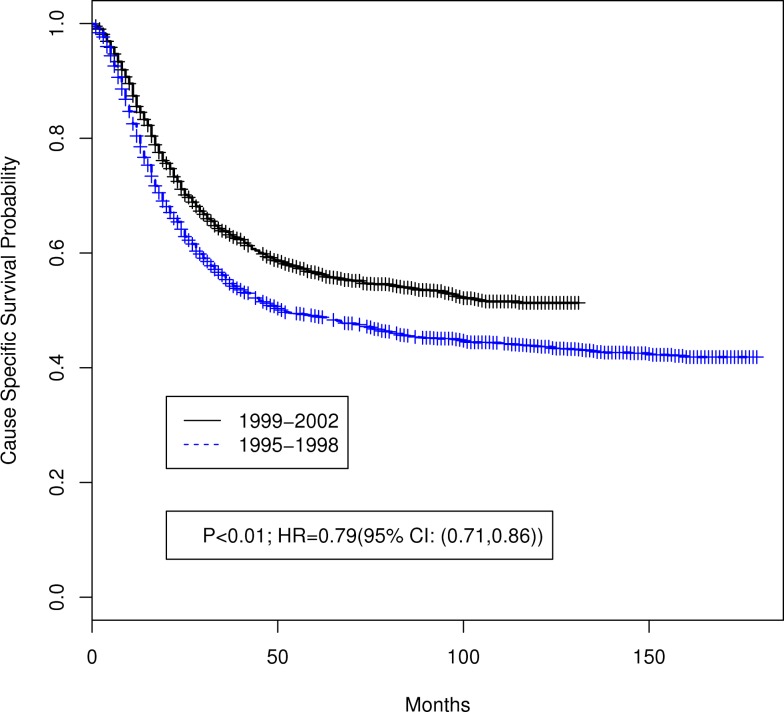
**Unadjusted Kaplan–Meier cause specific survival curves by 4-year time period (1999–2002 versus 1995–1998)**. Log-rank test *p* value and hazard ratio (HR) for death are shown.

Table 1 summarizes the distributions of patient, tumor, and treatment characteristics by the 4-year time periods (1995–1998 and 1999–2002). Note that small size subgroups were combined for statistical analyses (Table S1 in Supplementary material includes the original variable subgroups). We excluded “Tumor Size” because of the large proportion of unknown size cases. The results indicate that, with the exception of lymph node status and radiation type (*p* ≤ 0.05), there were no significant differences between the two time periods for patient, tumor, or treatment characteristics. The percentage of all patients who received “hysterectomy/exenteration/other surgery” was 28.8% (30.6% for 1995–1998, 27.1% for 1999–2002). Unadjusted analyses demonstrated that OS was significantly improved in 1999–2002 compared with 1995–1998 (*p* < 0.001; HR 0.81 with 95% CI: 0.74–0.88). The median OS time increased from 37 months (95% CI: 34–42 months) in 1995–1998 to 64 months (95% CI: 54–84 months) in 1999–2002 (Figure 2). We also found that CSS was significantly improved (*p* < 0.001; HR 0.79; 95% CI: 0.71–0.86). The median CSS increased from 52 months (95% CI: 44–71 months) in 1995–1998 to over 98 months (lower bound of the 95% CI, median not reached) in 1999–2002 (Figure 3). The percentage of non-cause specific death was 18% in 1995–1998 and was 17.5% in 1999–2002. Statistical methods that consider other causes of death as competing risks to cervical cancer deaths confirmed a significant difference in CSS between the two time periods (*p* < 0.0001) (data not shown).

**Table 1:** **Patient, tumor, and treatment characteristics by 4-year of diagnosis groups (*N* = 3517)**.

Variable	Year 1995–1998	Year 1999–2002	Total	*p*-Value
	# Patients (%)	# Patients (%)	
*N*	1758	1759	3517	
Age group				0.81
<41	372 (21.1)	360 (20.5)	732 (20.8)	
41–55	698 (39.7)	715 (40.7)	1413 (40.2)	
>55	688 (39.1)	684 (38.9)	1372 (39.0)	
Race				0.55
White	1239 (70.5)	1256 (71.4)	2495 (70.9)	
Non-white	519 (29.5)	503 (28.6)	1022 (29.1)	
Marital status				0.23
Married	747 (42.5)	712 (40.5)	1459 (41.5)	
Unmarried	1011 (57.5)	1047 (59.5)	2058 (58.5)	
FIGO stage				0.75
IB2	221 (12.6)	225 (12.8)	446 (12.7)	
II	877 (49.9)	855 (48.6)	1732 (49.3)	
III + IVA	660 (37.5)	679 (38.6)	1339 (38.0)	
Histology				0.59
Squamous cell carcinoma	1402 (79.7)	1383 (78.6)	2785 (79.2)	
Adenocarcinoma	168 (9.6)	186 (10.6)	354 (10.0)	
Other/unknown	188 (10.7)	190 (10.8)	378 (10.8)	
Lymph node status				0.006
Distant LN+	140 (7.96)	131 (7.45)	271 (7.71)	
LN−	872 (49.6)	945 (53.7)	1817 (51.7)	
Regional LN+	253 (14.4)	276 (15.7)	529 (15.0)	
Other/unknown	493 (28.0)	407 (23.1)	900 (25.6)	
Surgery extent				0.072
No surgery/incisional biopsy/unknown	1101 (62.6)	1153 (65.6)	2254 (64.1)	
Local ablation or excision	119 (6.8)	129 (7.3)	248 (7.1)	
Hysterectomy/exenteration/other surgery	538 (30.6)	477 (27.1)	1015 (28.8)	
Radiation type				0.026
Combined EBRT + brachytherapy	1089 (62.0)	1025 (58.3)	2114 (60.1)	
EBRT/brachytherapy/other	669 (38.0)	734 (41.7)	1403 (39.9)	

*Red color used to highlight statistically significant p-values*.

When stratified by age group, OS and CSS survival were significantly improved when comparing 1995–1998 with 1999–2002 in all age groups (Table 2). The HR (OS and CSS) of 1999–2002 compared to 1995–1998 was ≤0.85 in all age groups.

**Table 2:** **Survival of 1999–2002 compared to 1995–1998 stratified by age groups**.

Age group	Group size	OS		CSS		
		*p*-value by log-rank test	Hazard ratio 99-02 versus 95–98 (95% CI)	*p*-value by log-rank test	Hazard ratio 99-02 versus 95–98 (95% CI)	Proportion of non- CSD^a^ (%)
<41	732	<0.01	0.68 (0.55, 0.83)	<0.01	0.67 (0.54, 0.83)	7.2
41–55	1413	0.01	0.83 (0.72, 0.96)	0.02	0.83 (0.71, 0.97)	10.8
>55	1372	0.01	0.85 (0.75, 0.97)	<0.01	0.81 (0.70, 0.94)	27.4

*^a^“Non-CSD” is short for “Non-Cause Specific Death”. The “Non-CSD” patients were treated as censored in the cause specific survival (CSS) analysis*.

*Red color used to highlight statistically significant p-values*.

Table 3 shows the comparison of OS and CSS between 1995–1998 and 1999–2002 stratified by SEER regions. With the exception of Connecticut, Hawaii, and New Mexico, the HR of 1999–2002 to 1995–1998 in most regions (9/13 regions; 70%) was ≤0.92. In particular, in San Francisco–Oakland SMSA, Metropolitan Detroit, and Metropolitan Atlanta, the HRs for 1999–2002 relative to 1995–1998 were ≤0.73. In contrast, Connecticut, Hawaii, and New Mexico had HRs >1.00 (not statistically significant).

**Table 3:** **Survival of 1999–2002 compared to 1995–1998 stratified by SEER registries**.

SEER registry	Size	OS		CSS		
		*p*-value by log-rank test	Hazard ratio 99-02 versus 95–98 (95% CI)	*p*-value by log-rank test	Hazard ratio 99-02 versus 95–98 (95% CI)	Proportion of non- CSD^a^ (%)
San Francisco–Oakland SMSA	303	0.03	0.72 (0.53, 0.97)	0.05	0.71 (0.50, 1.00)	22.7
Connecticut	251	0.99	1.00 (0.73, 1.37)	0.33	0.84 (0.59, 1.20)	21.5
Metropolitan Detroit	374	<0.01	0.62 (0.48, 0.78)	<0.01	0.54 (0.41, 0.72)	15.4
Hawaii	125	0.57	1.14 (0.72, 1.81)	0.89	1.03 (0.61, 1.78)	28
Iowa	253	0.31	0.85 (0.61, 1.17)	0.27	0.82 (0.58, 1.17)	13.9
New Mexico	166	0.89	1.03 (0.69, 1.53)	0.89	1.03 (0.67, 1.57)	11.2
Seattle (Puget Sound)	264	0.32	0.85 (0.62, 1.18)	0.46	0.88 (0.63, 1.24)	15.8
Utah	125	0.11	0.68 (0.43, 1.09)	0.08	0.63 (0.38, 1.06)	13.7
Metropolitan Atlanta	255	0.05	0.73 (0.53, 1.00)	0.03	0.69 (0.48, 0.97)	14.6
Alaska	7	0.81	0.75 (0.07, 8.42)	0.81	0.74 (0.07, 8.42)	0
San Jose–Monterey	177	0.06	0.68 (0.45, 1.02)	0.05	0.64 (0.41, 1.00)	16.3
Los Angeles	1194	0.10	0.88 (0.76, 1.02)	0.36	0.92 (0.78, 1.09)	18.8
Rural Georgia	23	<0.01	0.06 (0.01, 0.29)	<0.01	0.10 (0.02, 0.50)	31.3

*^a^“Non-_CSD” is short for “Non-Cause Specific Death”. The “Non-CSD” patients were treated as censored in the cause specific survival (CSS) analysis*.

*Red color used to highlight statistically significant p-values*.

Cox regression models were used in order to examine changes in survival between 1995–1998 and 1999–2002, adjusted by other variables reported in the SEER database: race, marital status, FIGO stage, histology, lymph node status, surgery extent, and radiation type. Significant improvements in OS and CSS were observed after adjustment for all of the characteristics individually (Table 4). The HR for 1999–2002 compared to 1995–1998 was consistently 0.80 (95% CI: 0.74–0.88) for OS and 0.78 (95% CI: 0.71–0.86) for CSS when adjusted for each of these variables individually.

**Table 4:** **Survival comparisons of 1999–2002 versus 1995–1998 when adjusted by variables individually using Cox Model (*N* = 3517)**.

Variable	Size	OS				CSS			
		Estimate	SE	*p-*value	Hazard ratio (95% CI)	Estimate	SE	*p*-value	Hazard ratio (95% CI)
Race (ref = white)	2495								
Non-white	1022	0.015	0.048	0.757	1.015 (0.924,1.116)	−0.032	0.054	0.551	0.968 (0.872,1.076)
Year 1999–2002 (ref = 1995–1998)		−0.215	0.045	<0.001	0.807 (0.739,0.881)	−0.242	0.049	<0.001	0.785 (0.713,0.864)
Marital status (ref = married)	2058								
Unmarried	1459	0.225	0.045	<0.001	1.252 (1.146,1.368)	0.139	0.049	0.005	1.150 (1.044,1.266)
Year 1999–2002 (ref = 1995–1998)		−0.218	0.045	<0.001	0.804 (0.737,0.877)	−0.244	0.049	<0.001	0.783 (0.712,0.862)
Figo stage (ref = IB2)	446								
II	1732	0.359	0.082	<0.001	1.432 (1.219,1.683)	0.373	0.093	<0.001	1.452 (1.210,1.742)
III + IVA	1339	1.147	0.081	<0.001	3.150 (2.684, 3.697)	1.210	0.091	<0.001	3.352 (2.800, 4.014)
Year 1999–2002 (ref = 1995–1998)		−0.234	0.045	<0.001	0.791 (0.725,0.864)	−0.262	0.049	<0.001	0.769 (0.699,0.846)
Histology (ref = squamous cell carcinoma)	2785								
Adenocarcinoma	354	0.103	0.072	0.151	1.109 (0.963,1.276)	0.164	0.077	0.035	1.178 (1.012,1.371)
Other/unknown	378	0.238	0.069	0.0005	1.268 (1.109,1.451)	0.294	0.074	<0.001	1.342 (1.160,1.552)
Year 1999–2002 (ref = 1995–1998)		−0.216	0.045	<0.001	0.806 (0.739,0.880)	−0.244	0.049	<0.001	0.784 (0.713,0.863)
Lymph node status (ref = LN−)	1817								
Distant LN+	271	0.861	0.076	<0.001	2.366 (2.039,2.747)	0.991	0.081	<0.001	2.696 (2.300,3.160)
Regional LN+	529	0.250	0.066	<0.001	1.284 (1.129,1.460)	0.390	0.070	<0.001	1.477 (1.287,1.696)
Other/unknown	900	0.520	0.052	<0.001	1.683 (1.520,1.863)	0.543	0.058	<0.001	1.721 (1.536,1.929)
Year 1999–2002 (ref = 1995–1998)		−0.196	0.045	<0.001	0.822 (0.753,0.897)	−0.226	0.049	<0.001	0.798 (0.725,0.878)
Surgery extent (ref = no surgery/incisional biopsy/unknown)	2254								
Local ablation or excision	248	−0.411	0.092	<0.001	0.663 (0.553,0.794)	−0.443	0.104	<0.001	0.642 (0.524,0.788)
Hysterectomy/exenteration/other surgery	1015	−0.558	0.053	<0.001	0.572 (0.516,0.634)	−0.498	0.057	<0.001	0.607 (0.543,0.679)
Year 1999–2002 (ref = 1995–1998)		−0.230	0.045	<0.001	0.794 (0.728,0.867)	−0.256	0.049	<0.001	0.774 (0.703,0.852)
Radiation type (ref = combined EBRT + brachytherapy	2114								
EBRT/brachytherapy/other	1403	0.351	0.044	<0.001	1.421 (1.303,1.550)	0.376	0.049	<0.001	1.457 (1.324,1.603)
Year 1999–2002 (ref = 1995–1998)		−0.225	0.045	<0.001	0.799 (0.732,0.872)	−0.252	0.049	<0.001	0.777 (0.706,0.855)

*Red color used to highlight statistically significant p-values*.

We estimated the HR for levels of these characteristics, adjusted for time period, as shown in Table 4. For example, when adjusted by time period, the HR for OS for unmarried cases compared to married cases was 1.25 (95% CI: 1.15–1.37). Similarly, the OS HR was 1.43 (95% CI: 1.22–1.68) for FIGO stage II compared to IB2, and it was 3.15 (95% CI: 2.68–3.70) for FIGO stage III + IVA compared to IB2.

Multivariable Cox regression models were used to compare the OS and CSS between 1995–1998 and 1999–2002, adjusted simultaneously for all characteristics (Table 5: OS, Table 6: CSS). OS and CSS were significantly improved in 1999–2002 compared to 1995–1998 when adjusted for all factors. The HR for death in 1999–2002 compared to 1995–1998 was 0.78 for OS (95% CI: 0.71–0.85) and was 0.76 for CSS (95% CI: 0.69–0.84). Significance levels and HRs for categories within each variable are tabulated in Table 5 (OS) and Table 6 (CSS).

**Table 5:** **Best fitting Multivariable Cox model for overall survival (*N* = 3517, AIC = 31399)**.

Variable	Size	Parameter estimate	SE	*p*-value	Hazard ratio (95% CI)
Year 1999–2002 [ref =1995–1998 (*n*=1758)]	1759	−0.250	0.045	<0.001	0.779 (0.713,0.850)
Age [ref = <41 (*n* = 732)]					
41–55	1413	−0.079	0.064	0.219	0.924 (0.815,1.048)
>55	1372	0.221	0.063	<0.001	1.247 (1.102,1.411)
Marital status [ref = married (*n* = 2058)]					
Unmarried	1459	0.100	0.046	0.029	1.106 (1.010,1.210)
FIGO stage [ref = IB2 (*n* = 446)]					
II	1732	0.207	0.086	0.015	1.230 (1.040,1.455)
III + IVA	1339	0.834	0.088	<0.001	2.301 (1.938, 2.734)
Histology [ref = squamous cell carcinoma (*n* = 2785)]					
Adenocarcinoma	354	0.234	0.072	0.001	1.263 (1.096,1.456)
Other/unknown	378	0.308	0.069	<0.001	1.360 (1.187,1.558)
Lymph node status [ref = LN− (*n* = 1817)]					
Distant LN+	271	0.628	0.078	<0.001	1.874 (1.608, 2.184)
Regional LN+	529	0.271	0.068	<0.001	1.311 (1.146, 1.499)
Other/unknown	900	0.282	0.053	<0.001	1.326 (1.194,1.472)
Surgery Extent [ref = no surgery/incisional biopsy/unknown (*n* = 2254)]					
Local ablation or excision	248	−0.311	0.093	<0.001	0.733 (0.611,0.879)
Hysterectomy/exenteration/other surgery	1015	−0.482	0.060	<0.001	0.618 (0.549,0.694)
Radiation type [ref = combined EBRT + brachytherapy (*n* = 2114)]					
EBRT/brachytherapy/other	1403	0.409	0.046	<0.001	1.505 (1.375,1.646)

*Red color used to highlight statistically significant p-values*.

**Table 6:** **Best-fitting Multivariable Cox model for cause specific survival (*N* = 3517, AIC = 25951)**.

Variable	Size	Parameter estimate	SE	*p*-value	Hazard ratio (95% CI)
Year 1999–2002 [ref = 1995–1998 (*n*=1758)]	1759	−0.274	0.049	<0.001	0.760 (0.690,0.837)
FIGO stage [ref = IB2 (*n* = 446)]					
II	1732	0.314	0.096	0.001	1.369 (1.135,1.652)
III + IVA	1339	0.995	0.097	<0.001	2.706 (2.236, 3.275)
Histology [ref = squamous cell carcinoma (*n* = 2785)]					
Adenocarcinoma	354	0.318	0.078	<0.001	1.374 (1.179,1.601)
Other/unknown	378	0.352	0.075	<0.001	1.422 (1.227,1.647)
Lymph node status [ref = LN− (*n* = 1817)]					
Distant LN+	271	0.730	0.083	<0.001	2.075 (1.763, 2.444)
Regional LN+	529	0.384	0.073	<0.001	1.468 (1.272, 1.694)
Other/unknown	900	0.314	0.060	<0.001	1.369 (1.218,1.538)
Surgery Extent [ref = no surgery/incisional biopsy/unknown (*n* = 2254)]					
Local Ablation or excision	248	−0.365	0.104	0.0005	0.694 (0.565,0.852)
Hysterectomy/exenteration/other surgery	1015	−0.465	0.064	<0.001	0.628 (0.553,0.714)
Radiation type [ref = combined EBRT + brachytherapy (*n* = 2114)]					
EBRT/brachytherapy/other	1403	0.422	0.050	<0.001	1.525 (1.382,1.684)

*Red color used to highlight statistically significant p-values*.

A simulation study was conducted to test the robustness of these results under different assumptions for the proportions of patients receiving CCRT in the two time periods. For example, if we assumed that CCRT was given to 30% of the patients diagnosed in 1995–1998 and 70% of the patients diagnosed in 1999–2002, the simulation suggested that 52% of the time the log-rank tests for OS would not be significant. Alternatively, if we assumed that CCRT was given to 30% of the patients diagnosed in 1995–1998 and 100% of the patients diagnosed in 1999–2002, the simulation showed that 2% of the time the log-rank tests for OS would not be statistically significant (data not shown).

## Discussion

The 1999 NCI alert was based on five randomized trials showing improvement in OS with CCRT compared with radiation alone.

The Gynecologic Oncology Group (GOG) conducted a randomized controlled trial, GOG 123, for patients with bulky FIGO stage IB cervical cancers ≥4 cm in diameter (2). Patients were randomized to radiotherapy alone or combination chemoradiotherapy with weekly cisplatin. All patients had adjuvant extrafascial hysterectomy. Compared to the RT alone group, the chemoradiotherapy group had improved 4-year progression free survival (RR 0.51) and OS (RR 0.54).

The Radiation Therapy Oncology Group (RTOG) conducted a randomized controlled trial, RTOG 90-01, comparing extended field radiation therapy alone versus pelvic field chemoradiotherapy for women with FIGO stage IIB–IVA cervical carcinoma (3). Estimated 5-year OS and disease-free survival were both significantly improved with chemoradiotherapy (73 and 67%) compared to extended field radiation therapy alone (58 and 40%).

GOG 120 studied different chemoradiotherapy regimens in FIGO IIB–IVA cervical cancer patients (4). All patients received pelvic radiation therapy and intracavitary brachytherapy. Patients were randomized to one of three arms of concurrent chemotherapy: cisplatin alone versus cisplatin, fluorouracil and hydroxyurea versus hydroxyurea alone. Both cisplatin containing regimens were superior to hydroxyurea alone. Similarly, GOG 85 demonstrated the superiority of CCRT with cisplatin and fluorouracil over hydroxyurea alone for FIGO IIB–IVA cervical cancer patients (5). These two studies supported cisplatin-based chemotherapy for locoregional cervical cancer and added to the evidence for the overall efficacy ofCCRT.

Finally, the Southwest Oncology Group (SWOG) 8797 trial demonstrated that adjuvant CCRT improved overall and progression-free survival after radical hysterectomy and pelvic lymphadenectomy in patients found to have positive margins, positive parametria, or involved lymph nodes on pathology (6). Cisplatin-based chemoradiotherapy increased the 4-year progression-free survival to 80% compared to 63% for patients receiving radiation alone. Similarly, chemoradiation increased 4-year OS to 81% compared to 71% for radiation alone.

A recent meta-analysis including 13 trials that had unconfounded comparisons of chemoradiotherapy versus radiotherapy alone concluded that there was a 6% improvement in 5-year OS with chemoradiotherapy (HR = 0.81, *p* < 0.001) (9). Local and distant recurrences were also reduced and disease-free survival improved with chemoradiotherapy. The meta-analysis included the GOG 123 and SWOG 8797 trials in the main analysis, and it included the GOG 85, RTOG 90-01, and GOG 120 trials in a sensitivity analysis that supported the findings of the main analysis.

Our study similarly found an improvement in OS and CSS for the years 1999–2002 (CCRT era) compared with years 1995–1998 (RT era) in both unadjusted analyses (OS HR = 0.81, CSS HR = 0.79) and adjusted analyses (multivariable OS HR = 0.78, CSS HR = 0.76). Stratification by age group or geographic SEER region did not significantly alter these results. These results provide indirect evidence that the increased usage of CCRT in the era after the 1999 NCI alert may have improved OS and CSS for patients with IB2-IVA cervical cancer.

From the multivariable analyses, we note that HRs for death were significantly worse for age >55 versus ≤55, unmarried status versus married, FIGO stages II–IVA versus IB2, non-squamous histology versus squamous cell carcinoma, positive lymph nodes versus negative lymph nodes, biopsy only/no surgery versus local ablation/excision/hysterectomy/exenteration, and other radiation type versus combined EBRT + brachytherapy. Similarly, HRs for cause-specific death were significantly worse for FIGO stages II–IVA versus IB2, non-squamous histology versus squamous cell carcinoma, positive lymph nodes versus negative lymph nodes, biopsy only/no surgery versus local ablation/excision/hysterectomy/exenteration, and other radiation type versus combined EBRT + brachytherapy. These results are consistent with prior reports in the literature with respect to histology (13, 14), clinical stage, and pelvic nodal status (15). The literature is mixed regarding age as a prognostic factor, with some studies showing younger age as a poor prognostic factor and others showing the opposite effect or no effect of age when accounting for other variables (15–19). We found that age was not a significant factor for cervical cancer CSS on multivariable analysis.

Our confidence in attributing the improved survival during 1999–2002 to CCRT depends on the assumption that the rates of CCRT use were low between 1995 and 1998 and high between 1999 and 2002. While the primary analyses and outcomes are not contingent on any specific assumptions of percentages of CCRT use in the two time periods, the ability to attribute the outcomes to CCRT is affected by these percentages. In the best case scenario, where CCRT was given to 0% of the patients between 1995 and 1998 and 100% of the patients between 1999 and 2002, one could confidently attribute the significant improvements in survival in the latter time period to significant survival benefits of CCRT use. A simulation was performed that showed robustness of the overall study conclusions to moderate deviations (up to 30% per time period) in the percentages of patients receiving CCRT.

Data in support of our study assumptions and results come from a population-based cohort study from Ontario, Canada, which showed an increase in utilization of concurrent chemotherapy from <10% of RT cases between 1992 and 1998 to over 60% of RT cases between 1999 and 2001 (20). Consistent with this increased utilization of concurrent chemotherapy, the study reported an increase in 3-year OS from 58.6% in the 1992–1998 cohort to 69.8% in the 1999–2001 cohort among patients treated with primary RT (with or without chemotherapy).

One major limitation of using the SEER database for this study was that chemotherapy usage was not recorded for analysis in SEER*Stat. The SEER-Medicare linked database would contain detailed information about chemotherapy utilization, but approximately three-quarters of the women in our study were under 65 years old and would not have Medicare data available. Additionally, for postoperative patients, the SEER data were not sufficiently detailed to classify patients into cohorts that would meet the Sedlis criteria for postoperative RT alone (21, 22) versus the Peters criteria for postoperative CCRT (6). Specifically, data were not recorded in SEER*Stat for lymphovascular invasion for cervical cancer, depth of cervical stromal invasion, pathologic margin status for cervical cancer, and pathologic parametrial involvement. Inclusion of postoperative patients in the overall analyses more likely biased the results in favor of the null hypothesis because some of the patients in the 1999–2002 time period would have been appropriately treated with postoperative RT alone, thereby diluting the impact of those treated with CCRT.

A limitation of using the SEER database for the CSS analysis was that causes of death other than cervical cancer were treated as censored by the SEER database. This assumption could lead to bias if the cervical cancer deaths and other deaths were not independent. To account for this limitation, competing risk analyses were performed that confirmed the significant difference in CSS between the two time periods.

Other limitations include the retrospective nature of the study and lack of data on performance status, comorbidities, and other factors that may affect the decision to give concurrent chemotherapy. There may be unidentified differences in patient characteristics or non-chemotherapy treatments between the two time periods that could contribute to the observed survival differences. On the other hand, the study is strengthened by the large size of the study cohort and the narrow time periods studied (thereby diminishing the effects of stage migration or changes to radiation or surgical techniques).

In this study, we have not addressed the higher toxicity rates associated with CCRT since the SEER database does not include this information. Additional research is needed to discover new combinations of radiotherapy and systemic agents that can reduce toxicities and complications without compromising the survival benefits of CCRT.

In conclusion, since no other known changes in the demographics or management of locoregional cervical cancer occurred during the study time period, this study offers indirect evidence that the adoption of CCRT after the 1999 NCI alert improved both OS and CSS in patients with IB2-IVA cervical cancer. Furthermore, it appears that the recommendation to add concurrent chemotherapy to RT was adopted in a rapid time frame at least comparable to the adoption of clinical trial recommendations in other disease settings (23–25). To our knowledge, this is the first population-level study in the United States demonstrating improved survival for cervical cancer patients treated in the era of concurrent chemoradiation therapy.

## Conflict of Interest Statement

The authors declare that the research was conducted in the absence of any commercial or financial relationships that could be construed as a potential conflict of interest.

## Supplementary Material

The Supplementary Material for this article can be found online at http://journal.frontiersin.org/article/10.3389/fonc.2015.00081
