# Supplementary material for: Surveillance Epidemiology and End Results Analysis Demonstrates Improvement in Overall Survival for Cervical Cancer Patients Treated in the Era of Concurrent Chemoradiotherapy
Source: Front Oncol. 2015 Apr 13;5:81. doi: 10.3389/fonc.2015.00081 (PMC4394706; doi:10.3389/fonc.2015.00081)
Supplement: Supplementary file 1 [file Table_1.DOCX]

**Supplementary Table 1**. Patient, tumor, and treatment characteristics by 4-year of diagnosis groups (original table prior to regrouping for statistical analysis) (N=3517)

| **Variable** | **Year 1995-1998**  **# patients (%)** | **Year 1999-2002**  **# patients (%)** | **Total** |
| --- | --- | --- | --- |
| N | 1758 | 1759 | 3517 |
| Age Group |  |  |  |
| Less than 41 | 372 (21.1) | 360 (20.5) | 732 (20.8) |
| 41-55 | 698 (39.7) | 715 (40.7) | 1413 (40.2) |
| Greater than 55 | 688 (39.1) | 684 (38.9) | 1372 (39.0) |
|  |  |  |  |
| Race |  |  |  |
| White | 1239 (70.5) | 1256 (71.4) | 2495 (70.9) |
| Black | 249 (14.2) | 262 (14.9) | 511 (14.5) |
| American Indian/Alaska Native | 17 (1.0) | 25 (1.4) | 42 (1.2) |
| Asian/Pacific Islander | 248 (14.1) | 212 (12.1) | 460 (13.1) |
| Other/Unknown | 5 (0.3) | 4 (0.2) | 9 (0.3) |
|  |  |  |  |
| Marital Status |  |  |  |
| Single | 394 (22.4) | 473 (26.9) | 867 (24.7) |
| Married | 747 (42.5) | 712 (40.5) | 1459 (41.5) |
| Other/Unknown | 617 (35.1) | 574 (32.6) | 1191 (33.9) |
|  |  |  |  |
| Histology |  |  |  |
| Squamous Cell Carcinoma | 1402 (79.7) | 1383 (78.6) | 2785 (79.2) |
| Adenocarcinoma | 168 (9.6) | 186 (10.6) | 354 (10.1) |
| Other/Unknown | 188 (10.7) | 190 (10.8) | 378 (10.8) |
|  |  |  |  |
| Grade |  |  |  |
| Well differentiated, Grade I | 106 (6.0) | 88 (5.0) | 194 (5.4) |
| Moderately differentiated, Grade II | 501 (28.5) | 573 (32.6) | 1074 (30.5) |
| Poorly differentiated, Grade III | 691 (39.3) | 660 (37.5) | 1351 (38.4) |
| Undifferentiated/anaplastic,Grade IV | 48 (2.7) | 54 (3.1) | 102 (2.9) |
| Unknown | 412 (23.4) | 384 (21.8) | 796 (22.6) |
|  |  |  |  |
| FIGO Stage |  |  |  |
| IB2 | 221 (12.6) | 225 (12.8) | 446 (12.7) |
| II | 877 (49.9) | 855 (48.6) | 1732 (49.3) |
| III | 552 (31.4) | 574 (32.6) | 1126 (32.0) |
| IVA | 108 (6.1) | 105 (6.0) | 213 (6.1) |
|  |  |  |  |
| Tumor Size |  |  |  |
| 4cm or less | 227 (12.9) | 264 (15.0) | 491 (14.0) |
| Greater than 4cm | 787 (44.8) | 865 (49.2) | 1652 (47.0) |
| Unknown | 744 (42.3) | 630 (35.8) | 1374 (39.0) |
|  |  |  |  |
| Lymph Node Status |  |  |  |
| LN Neg | 872 (49.6) | 945 (53.7) | 1817 (51.7) |
| Regional LN+ | 253 (14.4) | 276 (15.7) | 529 (15.0) |
| Distant LN+ | 140 (7.96) | 131 (7.45) | 271 (7.71) |
| Other/Unknown | 493 (28.0) | 407 (23.1) | 900 (25.6) |
|  |  |  |  |
| Surgery Extent |  |  |  |
| No Surgery/Incisional Biopsy/Unknown | 1101 (62.6) | 1153 (65.6) | 2254 (64.1) |
| Local ablation or excision | 119 (6.8) | 129 (7.3) | 248 (7.1) |
| Hysterectomy, Any Type | 406 (23.1) | 435 (24.7) | 841 (23.9) |
| Exenteration/Other Surgery | 132 (7.5) | 42 (2.4) | 174 (5.0) |
|  |  |  |  |
| Radiation Type |  |  |  |
| EBRT | 611 (34.8) | 655 (37.2) | 1266 (36.0) |
| Brachytherapy | 44 (2.5) | 56 (3.2) | 100 (2.8) |
| Combined EBRT+Brachytherapy | 1089 (62.0) | 1025 (58.3) | 2114 (60.1) |
| Other Radiation | 14 (0.8) | 23 (1.3) | 37 (1.1) |
